# Experimental hypothermia by cold air: a randomized, double-blind, placebo-controlled crossover trial

**DOI:** 10.1186/s13049-025-01331-4

**Published:** 2025-01-31

**Authors:** Ane M. Helland, Sigurd Mydske, Jörg Assmus, Guttorm Brattebø, Øystein Wiggen, Haakon K. Kvidaland, Øyvind Thomassen

**Affiliations:** 1 Department of Clinical Medicine, University of Bergen, Storgata 33A, 0103 Bergen, Oslo, Norway; 2 Mountain Medicine Research Group, The Norwegian Air Ambulance Foundation, Bergen, Norway; 3 Department of Anesthesia and Intensive Care, Haukeland University Hospital, Bergen, Norway; 4 Norwegian National Advisory Unit On Emergency Medical Communication, Haukeland University Hospital, Bergen, Norway; 5 Department of Health Research, SINTEF Digital, Trondheim, Norway; 6 Department of Pediatrics, Haukeland University Hospital, Bergen, Norway

**Keywords:** Accidental hypothermia, Thermoregulation, Shivering, Protocol, Prehospital, Emergency medicine, Mountain medicine

## Abstract

**Background:**

Accidental hypothermia is associated with high morbidity and mortality. Research on treatment strategies for accidental hypothermia is complicated by the low incidence and heterogeneous patient population. We have developed a new method for clinical trials of experimental hypothermia, to enable further studies of active rewarming. If cold ambient air is effective as a cooling method, this would mimic the most frequent clinical setting of hypothermic patients and provide a feasible cooling method for field studies. We aimed to induce mild hypothermia in healthy volunteers by exposure to cold ambient air, and tested the hypothesis that drug-induced suppression of endogenous thermoregulation would be required.

**Methods:**

In a randomized, double-blind, crossover design, 15 healthy volunteers wearing wet clothes were put in a windy climate chamber set to 5 °C. Each participant completed the experimental procedure twice, once receiving active drugs (meperidine and buspirone) and once receiving placebo. The experiments were separated by a one-week wash-out period. Primary outcome was core temperature at termination, defined as 3 h of exposure or 35 °C. The between-groups difference was assessed using analysis of covariance (ANCOVA) with left censoring (Tobit model) and individual random intercept. Secondary outcomes were trajectory of core temperature and reduction of shivering.

**Results:**

At termination, the active drug vs placebo group differed in temperature by 1.4 °C. With adjustment for the removal of participants reaching 35 °C, the estimated mean difference was 1.7 °C (1.4–2.0, *p* < 0.001). Shivering was effectively reduced, but not completely inhibited by the drug regimen, and core temperature declined at a rate of − 0.82 °C per hour.

**Conclusion:**

The novel protocol utilizing cold air as a cooling method and drug-induced suppression of endogenous thermoregulation, is effective and enables future research projects. We have provided suggestions for minor alterations.

***Trial registration*:**

EudraCT ID 2023–506020-81–00.

## Introduction

Accidental hypothermia increases morbidity and mortality in many patient groups [1–5]. Only limited evidence supports current treatment guidelines, which are mainly based on case reports, retrospective registry studies, and expert opinions [6, 7]. By expanding our research methodologies we can provide more certain evidence regarding the management of accidental hypothermia. Conducting clinical trials on hypothermic patients is ethically questionable and would require large sample sizes due to the heterogenicity of this patient group. Studying volunteer research participants is the best alternative. To induce hypothermia in healthy volunteers we hypothesized the need for two interventions: a source of cooling and suppression of the endogenous mechanism of thermoregulation.

In experimental studies of hypothermia, core temperature reduction has been facilitated by cold intravenous (iv.) saline infusions; [8–17] cold-water immersion; [18–25] or by combinations of iv. infusions, forced air blankets, and/or water mattresses [26–37]. However, our clinical experience, supported by studies, suggests exposure to cold air to be the most frequent etiology of accidental hypothermia [38]. Thus, an experimental design in which participants are exposed to cold ambient air would better resemble the clinical setting of most hypothermic patients, and provide data more applicable to patients in the field.

To facilitate core temperature reduction in healthy volunteers, it may be necessary to eliminate shivering. This physiologic response to cold exposure produces considerable endogenous heat, counteracting the desired heat loss. Additionally, shivering is a disruptive factor in studies of hypothermia because the heat produced varies greatly between individuals, and may hide potential effects of exogenous rewarming techniques [18, 39, 40]. Inhibition of shivering would provide more standardized conditions for research purposes. Meperidine is a synthetic opioid that effectively inhibits shivering at lower equianalgesic dosages compared to other opioids. It is the drug most frequently used in previous studies of hypothermia in awake healthy volunteers [9, 14, 16–27, 33, 41, 42]. Moreover, meperidine combined with the anxiolytic buspirone exhibits a synergistic effect on shivering, reducing the opioid dose required [33]. In addition to suppressing shivering, meperidine affects the threshold for vasoconstriction, further enhancing the cooling effect [43].

We have developed a protocol tailored to a cold ambient air setting, designing a dosage scheme of meperidine and buspirone to adequately suppress shivering. In the present study, we aimed to evaluate whether our novel protocol for experimental hypothermia using cold ambient air would effectively reduce the core temperature of healthy volunteers. We hypothesized that inhibition of endogenous thermoregulation would be required. The primary outcome was core temperature at termination of the experiment. Secondary outcomes were temperature trajectory and inhibition of shivering.

## Method

### Study design

We conducted a randomized, double-blind, placebo-controlled, crossover, phase II clinical drug trial at Haukeland University Hospital, Bergen, Norway, in November 2023. The investigational medicinal products were a combination of meperidine and buspirone, compared to placebo*.* Participants were cooled down twice, once with the use of active drugs and once with placebo, serving as their own control. Each participant was tested at the same time of day, for each of their study days, to avoid circadian effects on body temperature. The two visits were scheduled one week apart to avoid carry-over effect, and to allow adequate wash-out of meperidine.

## Selection of participants

We enrolled 16 healthy volunteers of 18–40 years of age, with body mass index (BMI) < 30 kg/m^2^. Female participants provided a urinary sample to exclude pregnancy. Participants were instructed to consume a light meal on each day of experiment; to not use nicotine products at the study site; and to refrain from caffeine, alcohol, grapefruit juice, and strenuous exercise during the 24 h before each experiment. The participants received no payment.

## Interventions

### Climate chamber

Active cooling was performed in a refrigerated container, set at 5 °C, measuring 2.3 × 5.5 × 2.3 m. The room temperature and humidity were measured every minute by a logger (OMEGA OM-CP-MICRORHTEMP, Stamford, USA), which was placed on the floor behind the participant’s head. Participants were instructed to lie in the supine position on isolated sleeping pads (Therm-a-Rest Z-lite, R-value 2.0). Air fans (Ryobi 18 V R18F5-0) were placed at ground level next to the participants’ feet, and the wind was measured every 20 min by handheld equipment (Testo, Black Forest, Germany) at the thoracic level.

### Study drugs

A single per os (po.) dose of 30 mg buspirone or placebo was given 60 min prior to active cooling. Meperidine or placebo (saline) was administered in an initial bolus of 1 mg/kg iv., titrated by dividing the dose into five aliquots, each given at 2-min intervals during the last 10 min prior to entering the climate chamber. In the climate chamber, scheduled boluses of 0.5 mg/kg were administered every 30 min. In the event of break-through shivering, an additional “unscheduled” bolus of 0.25 mg/kg was administered. The minimum interval between boluses was set to 5 min. To avoid exceeding a safety limit of 4 mg/kg, no more than two unscheduled additional bolus doses could be administered. After the experiment, we measured normeperidine, a metabolite of meperidine. Drugs and dosages were decided based on a systematic search of the literature (manuscript in preparation).

Identically coated buspirone and placebo tablets were delivered by Kragerø Tablettproduksjon to Bergen Hospital Pharmacy, which stored, prepared, labelled, and accounted for both the po. and iv. study drugs. The study drugs were organized in kits, and the content (active drugs or placebo) was determined by a computer-generated randomized list. Each kit contained a sealed envelope containing blind-break information.

### Preparation

Instrumentation was conducted under thermoneutral indoor conditions. The participants received iv. cannulas for drug administration, transnasal esophageal probes for temperature measurements, a facemask to assess VO_2_, and were connected to equipment for monitoring vitals. Baseline measurements were recorded after 10 min of relaxing in supine position on a portable stretcher. Thereafter, participants were dressed in cotton sweatshirts and sweatpants soaked in cold water, compressed by hand to remove excess water. Participants wore dry mittens, hats, socks, and shoe covers to minimize discomfort and risk of frostbite, and diapers due to anticipated cold-induced diuresis.

## Experimental procedure

**Fig. 1:**
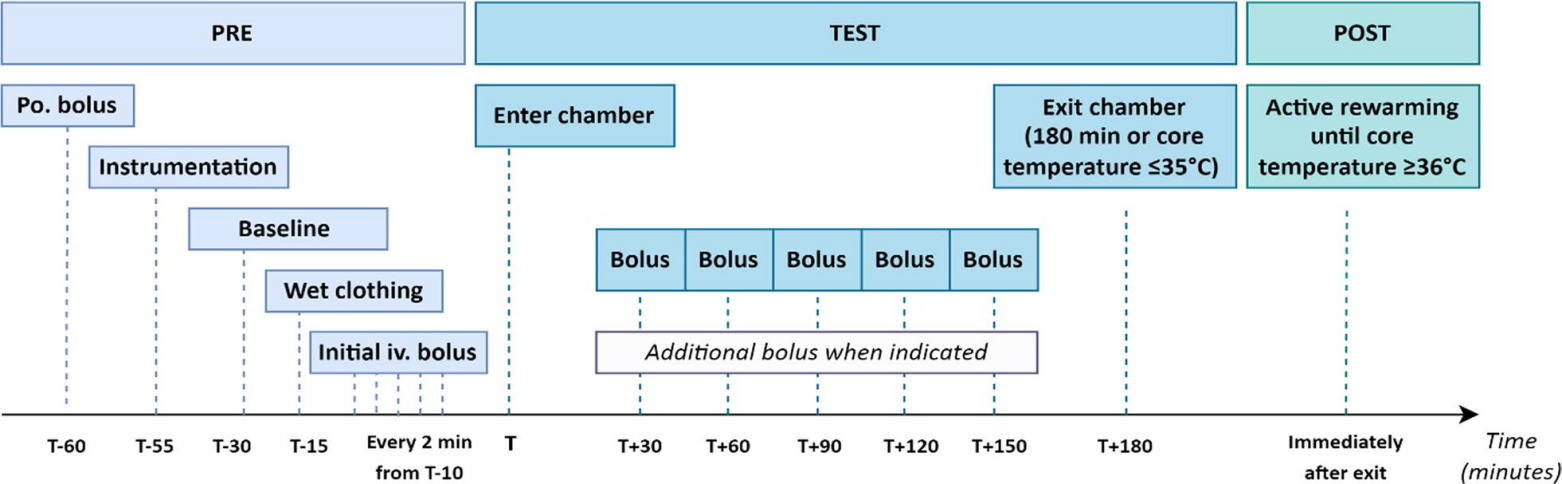
Timeline. Overview of the timeline on each study day. Includes the preparation phase, the experimental phase, and the rewarming phase. *T* = time on entrance to climate chamber

The participants walked into the climate chamber and were cooled until reaching a core temperature of 35 °C, or for a maximum of 3 h (Fig. 1).

## Measurements

### Temperature

Temperature was measured every minute using a transnasal esophageal probe inserted to the level of the heart, connected to a Corpuls3 monitor (Corpuls, Kaufering, Germany).

### Shivering

Oxygen consumption (VO_2_) was measured to indicate shivering, which was defined as a sustained (> 1 min) increase of ≥ 30% from baseline [10, 12]. Breath-by-breath measurements were obtained using the K5 portable metabolic system (CosMed, Rome, Italy), with OMNIA software v.2.2, and Long-Range Bluetooth 2.1 or USB cables for data transfer, calibrated according to the manufacturer’s specifications. The participants wore K5 face masks (CosMed, Rome, Italy) fixed in position by adequate headgear (Hans Rudolph Inc., Kansas City, USA).

### Safety

To ensure that participants remained within predefined safe ranges, we measured heartrate, blood pressure, oxygen saturation, and ECG using the Corpuls3 monitor. We monitored sedation by Richmond Agitation-Sedation Scale, respiratory rate by K5 described above, skin temperature by iButton® (Maxim integrated products Inc., Whitewater, USA), and normeperidine values. After extrication, participants were actively rewarmed in an infrared sauna until core temperature returned to 36 °C.

## Outcomes

The primary outcome was defined as core temperature at termination of experiment, comparing active drugs to placebo. Termination was defined by reaching an esophageal temperature of 35 °C, or active cooling exceeding 3 h. Secondary outcomes were trajectory of temperature and inhibition of shivering.

## Analysis

For sample size calculation, we defined a clinically significant difference between groups (active drugs vs. placebo) as a temperature difference of 0.5 °C at experiment termination. We assumed a standard deviation of 0.5 °C for interindividual variation, and 0.1 °C for intraindividual variation. In all calculated scenarios, 14 participants would yield a power of ≥ 90%, with a two-sided t-test significance level of 5%.

We used descriptive methods to characterize the sample. The between-groups difference in temperature at termination (primary endpoint) was assessed using the analysis of covariance (ANCOVA) with left censoring at 35 °C (Tobit model) and individual random intercept. This model takes into account the truncation of the temperature distribution at 35 °C (termination criteria) and dependent observations introduced by the cross-over design (two observations per participant). The carry-over effect of the cross-over was assumed to be 0 because of the sufficient wash-out period. We calculated the odds ratio for reaching 35 °C, and used the Haldane-Ascombe correction for the 95% confidence interval (CI) since no shivering participant reached 35 °C [44]. The difference between groups in trajectory of temperature and shivering (VO_2_) were assessed using the ANCOVA with individual random intercept at each time-point and the mean (95% CI) of the observed values*.* Shivering was also assessed based on the percentage of VO_2_ measurements exceeding baseline value by 30%. The significance level was set to 0.05. We used R 4.4.0 for data handling and computations, and MATLAB (The Mathworks Inc. Natick, MA) for graphics [45]. All analyses were performed on all collected data conducted according to protocol. No imputation methods were employed to handle missing data, except for esophageal temperature where single missing values and obviously erratic measurements (temperature < 34 °C, or a change of > 0.3 °C per minute) were replaced by the average of the 5 previous and 5 following measurements. We aligned the time of measurements between devices using the mean of the measured values per minute.

## Results

**Fig. 2:**
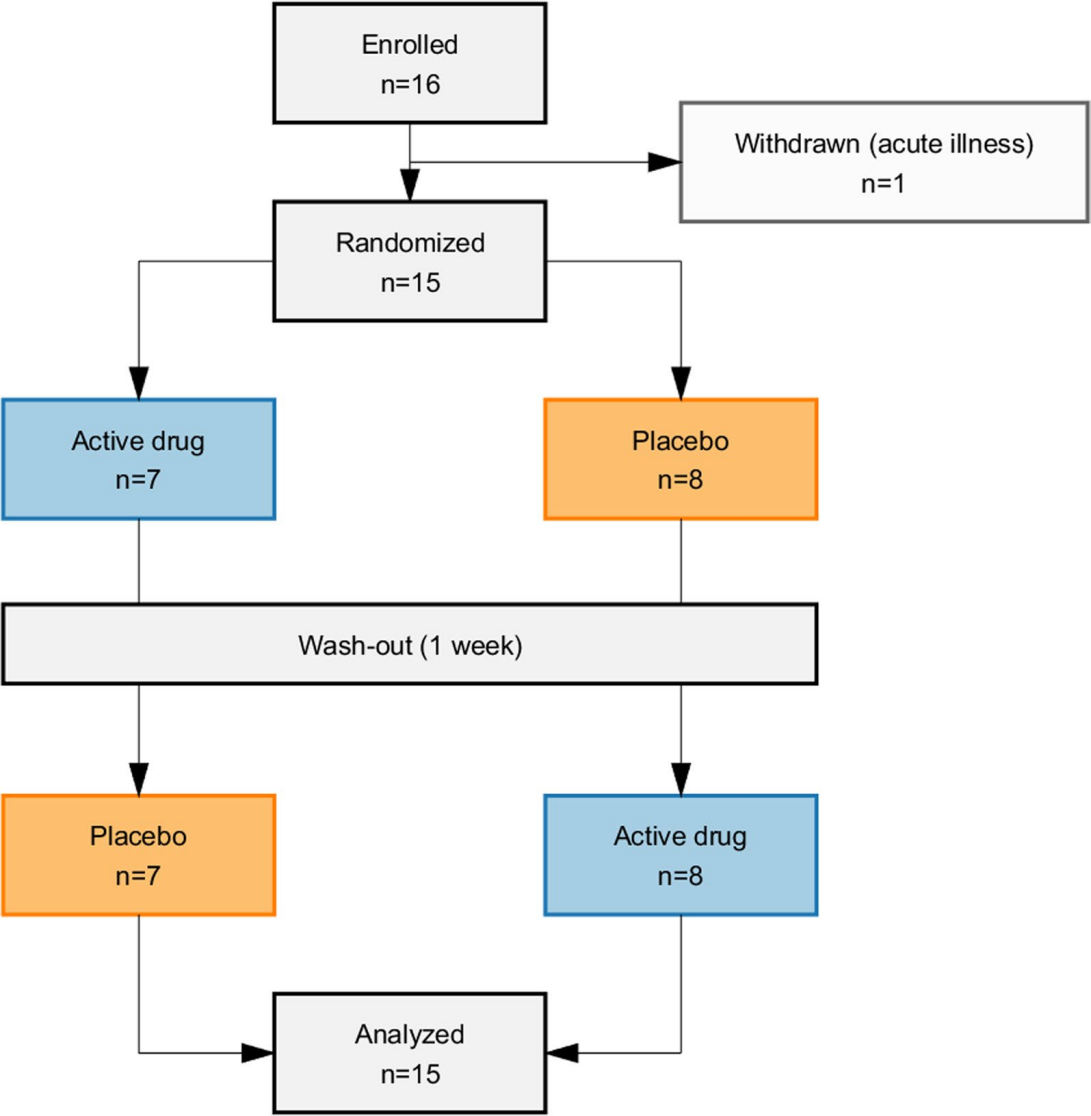
Flowchart of study protocol. One participant withdrew the day before the trial due to acute illness

15 participants (for characteristics, see Table 1) completed both visits and received the intended treatment in a randomized order (Fig. 2). The trial was completed in two weeks as scheduled, with no blind break.

**Table 1:** Participant- and chamber characteristics

*Participants*	
Age in years, median [min, max]	21 [19, 39]
Female sex, n (%)	7 (47%)
BMI in kg/m^2^, mean [min, max]	24.5 [20.8, 29.3]
*Climate chamber*	
Temperature, °C, median (min, max)	5.9 (5.5, 6.8)
Humidity, %, median (min, max)	88.4 (84.8, 90,5)
Wind, m/s, median (min, max)	1.8 (0.9, 2.8)

**Fig. 3:**
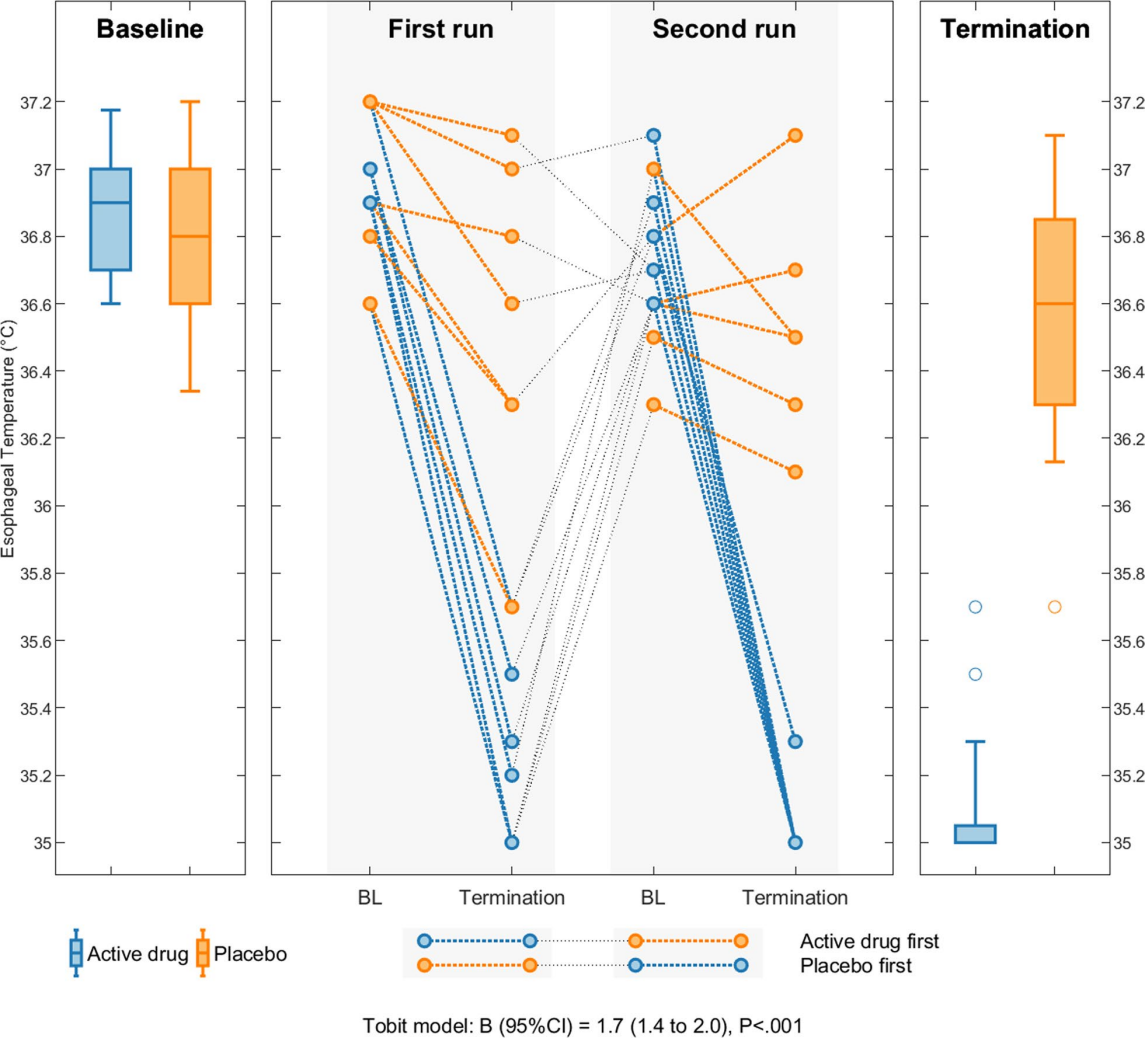
Core temperature. Esophageal temperature at baseline vs. termination in the active drug (blue) and placebo (orange) group

The temperature at termination differed by 1.4 °C between the active drug and the placebo group. After adjustment for the removal of participants reaching 35 °C, the estimated difference between groups was 1.7 °C (95% CI 1.4 °C to 2.0 °C, *P* < 0.001) (Fig. 3). The odds ratio of 55 (95% CI 3 to 1114) indicates that the group receiving active drugs had 55-times higher odds of reaching 35 °C.

**Fig. 4:**
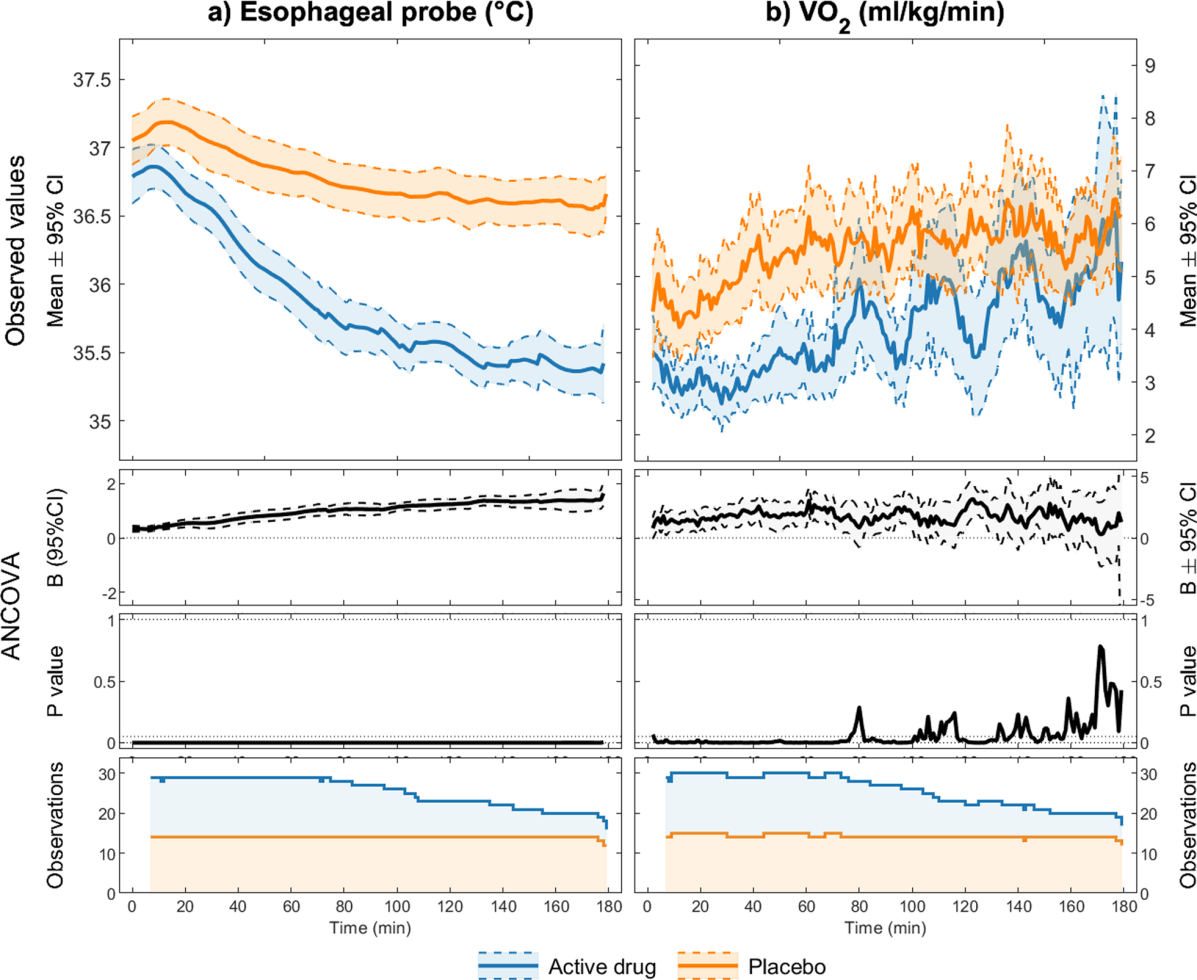
Temperature trajectory and shivering. Trajectories of esophageal temperature (**a**) and shivering (VO2) (**b**), with 95% CI and P values for the differences between the active drugs (blue) vs. placebo (orange) group. The numbers of observations for all time-points are shown at the bottom. X-axis shows time from entrance into the climate chamber

Figure 4 presents the trajectory of temperature change for both groups. Upon entering the climate chamber, we could see an increase in core temperature from baseline in the placebo group only. For participants receiving active drugs, core temperature declined at a rate of 0.82 °C/ hour. 10 of the 15 participants receiving active drugs reached 35.0 °C within the allotted timeframe, with a median time to termination of 1.78 h (min. 1.23 h, max. 3 h).

Shivering occurred much more frequently in the placebo group compared to the active drugs group (Table 2). Figure 4 illustrates the difference of mean VO_2_ in the active drug vs. placebo group. This difference was significant during the first 80 min of the cooling phase, but was no longer significant at the end of the experiment.

**Table 2:** Shivering indicated by increase in oxygen consumption

*Shivering (% of VO*_*2*_* measurements* ≥ *30% from baseline)*	
Active drugs, mean (min, max)	3.2 (0.3, 18.7)
Placebo, mean (min, max)	59.7 (18.7, 83.7)

**Fig. 5:**
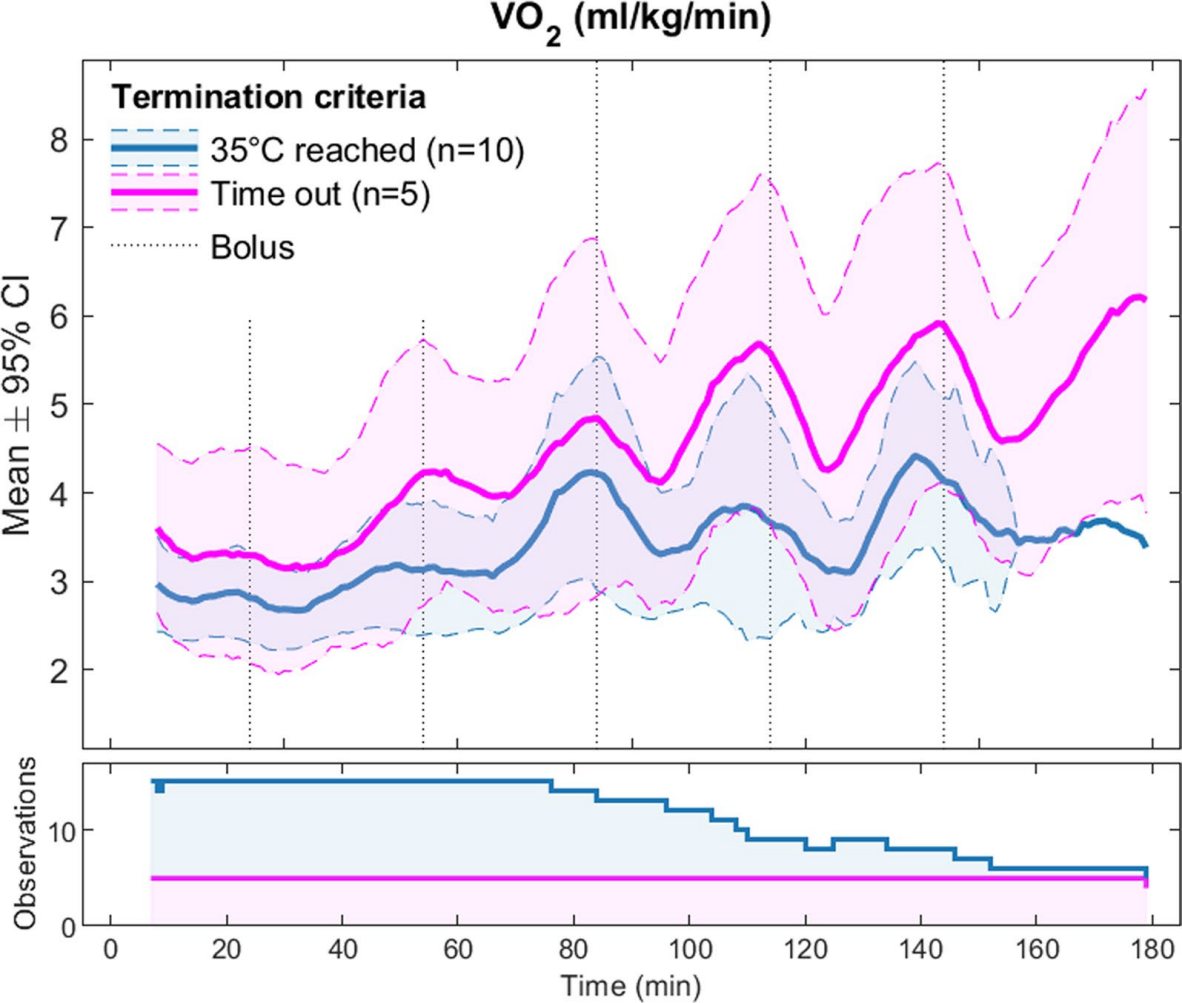
Shivering in the group receiving active drugs. The participants receiving shiver-inhibiting drugs is here split into two groups: those who reached 35 °C within the allotted timeframe (blue), and those who did not (pink). The number of observations in each group at each time-point is presented below. The stapled vertical lines indicate the timing of maintenance boluses

Figure 5 displays only the group receiving active drugs. It shows an increase of VO_2_ during the minutes preceding each scheduled bolus dose. The participants receiving active drugs are divided in two groups; participants reaching 35 °C within the alotted time frame and participants who did not. The participants reaching 35 °C had a lower oxygen consumption (VO_2_). This difference was present already from the start of cooling, and persisted throughout the experiment.

The esophageal temperature data from the placebo run of one participant was excluded from analysis, due to a technical error in measurement.

There was no discontinuation of study interventions due to safety issues, and Normeperidine values were within the pre-set safe limit.

## Discussion

In the present study, we demonstrated the possibility of using exposure to cold ambient air to induce mild hypothermia in volunteer research participants. Our results confirmed our hypothesis that endogenous thermoregulating mechanisms must be inhibited to facilitate adequate reduction of core temperature.

In general, shivering was suppressed by the selected drug regimen, and the inhibition of endogenous heat production enabled core temperature reduction. The lack of a significant difference in shivering between groups during the latter part of the experiment, was partly due to the removal of participants in the active drugs group who reached 35 °C. The participants who reached 35 °C had lower VO_2_ values and steeper cooling rates. Upon their removal, the remaining pool of participants may not have been completely shiver inhibited, increasing the mean VO_2_ at this time. To accurately measure a loosely defined mechanism is difficult. Shivering is described as involuntary, rapid, repeated contractions of skeletal muscle [46]. At rest, increased oxygen consumption (VO_2_) from baseline indicates energy expenditure due to thermogenesis, and thus provides an estimated measure of shivering. The lack of significant difference may also be explained by the sensitive equipment used for breath-by-breath VO_2_ measurement; the large variability in VO_2_ measurements yielded large spread in the results, with correspondingly large confidence intervals.

Baseline temperatures were similar between the two groups, but we observed that the active and placebo groups differed slightly upon entrance into the chamber. This was likely due to heat generation triggered by the cold stimulus of putting on wet clothes. During the short period between baseline and entrance into chamber, the placebo group was able to thermoregulate, generating heat by shivering and reducing heat loss by vasoconstriction, while the group receiving active drugs could not. Both groups exhibited a small increase of temperature during the first minutes after entrance, which could be attributed to shifts in body position and heat generated by skeletal muscles during the walk from the preparation room to the climate chamber (20 m).

No previous study has described cooling rates in a cold ambient environment when endogenous thermoregulating mechanisms is suppressed. During cold water immersion, reported cooling rates have been − 3.4 °C/h for immersion in 12 °C water, [19] and − 1.7 °C/h. for immersion in 17 °C water [25]. For iv. infusion in 4 °C ringer solution, a cooling rate of − 2.4 °C has been documented [16]. As expected, cold ambient air provides a lesser cold stimulus, yielding a lower cooling rate of − 0.8 °C/h. When shivering was inhibited, the temperature trajectory followed a predictable pattern with a gradual decline towards hypothermia.

Five participants receiving active drugs did not drop below 35 °C within the allotted timeframe. These participants did not differ in demographics from the group reaching hypothermia. Notably, these five participants had higher VO_2_ values present already from the start of experiment, before opioid administration. This suggests that the difference could be due to inherent individual attributes of thermoregulation, including aspects such as individual shivering thresholds or heat-generating abilities, rather than differences in drug distribution and elimination. In general, the shivering data greatly varied between individuals, in accordance with previous studies, [47, 48] underlining the need for shivering suppression to generate standardized research conditions.

We observed a pattern of increasing VO_2_ during the minutes preceding each next scheduled bolus, starting after one hour in the climate chamber (Fig. 5). This pattern may be due to rapid elimination of meperidine, resulting in break-through shivering. This insufficiency in the dosing scheme could be managed by either increasing the doses or reducing the interval between boluses. The doses were selected based on a review of existing literature [18–25, 33]. Since the initial bolus dose and first scheduled maintenance dose were sufficient for suppressing shivering, we rather suggest decreasing the time interval between scheduled boluses from every 30 min to every 25 min in future protocols. These spikes in VO_2_ preceding each maintenance bolus probably results in a heat debt for the next couple of minutes. Reducing these spikes would likely accelerate the heat loss and reduce the time needed to reach mild hypothermia, thus limiting the participants’ exposure time.

Our protocol was designed to reduce core temperature in volunteers, to facilitate further research on the effect of different active rewarming devices and other treatment options for hypothermic patients. Compared to immersion experiments, our approach to temperature reduction requires less resources, with simplified monitoring and logistics, and thus expands the possibilities for mountain field research. The cooling method replicates typical real-life conditions experienced by patients in a pre-hospital setting, the yielding results will therefore be more applicable to the clinical setting of most patients. Due to the inhibition of shivering, the protocol can also serve as a human model for moderate/severe hypothermia.

One could argue that this non-shivering state is otherwise not clinically relevant, because real patients can generate heat by shivering. However, in quite a few settings, a patient’s thermoregulating capacity is no longer intact or has surpassed its limits. This includes patients with moderate (28–32 °C) or severe (< 28 °C) hypothermia, head injuries, hypoglycemia, [49, 50] hypothyroidism, [51] fatigue, [52] intoxication, or iatrogenic causes, such as opioid administration [29, 33] by health personnel. Our results suggests that this population may experience steeper cooling rates, not only if submerged or buried in snow, but also in cold ambient temperatures. Health personnel administering opioids or other drugs should be aware of this potential effect on thermoregulation. When encountering a non-shivering patient in a cold environment, one may mistakenly perceive the patient as “not very cold”. We would argue the opposite: if a patient is not benefiting from heat production via shivering, their temperature could be decreasing more rapidly than that of shivering patients.

## Limitations

Interpretation of the present results is limited to the investigated condition (set temperature, wind, and wet clothing), and to a healthy population of similar age and BMI. The response to temperature manipulation may differ between healthy volunteers with intact resources versus patients. There might be a selection bias towards volunteers more tolerant of cold environments than the average person; however, they might be representative of the population spending time outdoors, and thus more frequently suffer from accidental hypothermia.

Inhibition of shivering alters the body’s response to cold. Our selected drugs do not affect thermoregulation solely by inhibiting shivering; thermoregulation is also affected by vasodilation due to histamine release by meperidine, [53, 54] and by activation of 5-HT1A (serotonin) receptors by buspirone [55, 56]. Notably, in contrast to other opioids, meperidine has double the effect on shivering thresholds compared to on vasodilation [57]. The mechanism for thermoregulation is ancillary to our primary objective of reducing core temperature. However, when applying the method in further studies and extrapolating results to patients, all effects of the drugs should be considered.

Although this study was blinded, in some cases it became evident to both the participants and investigators whether active or placebo drugs were administered, due to presence or absence of shivering, or the clinical effects of opioids.

## Conclusion

This novel protocol utilizing cold air as a cooling method, and a tailored drug regimen to suppress endogenous thermoregulation, effectively reduces core temperature in healthy volunteers. We have provided suggestions for minor alterations of the intervals between scheduled maintenance doses.

## Data Availability

The complete study protocol and the deidentified data subset for this investigation is available upon reasonable request if ethical approval is granted. Contact corresponding author.

## Abbreviations

ANCOVA: Analysis of covariance
iv.: Intravenous
BMI: Body mass index
po.: Per os
VO_2_: Oxygen consumption
CI: Confidence interval
